# F_420_H_2_-Dependent Degradation of Aflatoxin and other Furanocoumarins Is Widespread throughout the *Actinomycetales*

**DOI:** 10.1371/journal.pone.0030114

**Published:** 2012-02-27

**Authors:** Gauri V. Lapalikar, Matthew C. Taylor, Andrew C. Warden, Colin Scott, Robyn J. Russell, John G. Oakeshott

**Affiliations:** Ecosystem Sciences, Commonwealth Science and Industrial Research Organisation, Canberra, Australian Capital Territory, Australia; Louisiana State University and A & M College, United States of America

## Abstract

Two classes of F_420_-dependent reductases (FDR-A and FDR-B) that can reduce aflatoxins and thereby degrade them have previously been isolated from *Mycobacterium smegmatis*. One class, the FDR-A enzymes, has up to 100 times more activity than the other. F_420_ is a cofactor with a low reduction potential that is largely confined to the *Actinomycetales* and some *Archaea* and *Proteobacteria*. We have heterologously expressed ten FDR-A enzymes from diverse *Actinomycetales*, finding that nine can also use F_420_H_2_ to reduce aflatoxin. Thus FDR-As may be responsible for the previously observed degradation of aflatoxin in other *Actinomycetales*. The one FDR-A enzyme that we found not to reduce aflatoxin belonged to a distinct clade (herein denoted FDR-AA), and our subsequent expression and analysis of seven other FDR-AAs from *M. smegmatis* found that none could reduce aflatoxin. Certain FDR-A and FDR-B enzymes that could reduce aflatoxin also showed activity with coumarin and three furanocoumarins (angelicin, 8-methoxysporalen and imperatorin), but none of the FDR-AAs tested showed any of these activities. The shared feature of the compounds that were substrates was an α,β-unsaturated lactone moiety. This moiety occurs in a wide variety of otherwise recalcitrant xenobiotics and antibiotics, so the FDR-As and FDR-Bs may have evolved to harness the reducing power of F_420_ to metabolise such compounds. Mass spectrometry on the products of the FDR-catalyzed reduction of coumarin and the other furanocoumarins shows their spontaneous hydrolysis to multiple products.

## Introduction

Aflatoxins are complex aromatic compounds containing α,β-unsaturated lactone moieties that are produced most notably by *Aspergillus flavus* and *Aspergillus parasiticus* and have high chronic toxicity and carcinogenicity in animals, including humans [1]. Although relatively recalcitrant to biodegradation, aflatoxins are known to be degraded by some species from the order *Actinomycetales*, specifically *Nocardia corynebacteriodes* [2], *Rhodococcus erythropolis*, *Mycobacterium fluorantheniorans* sp. Nov DSM44556^T^ [3], [4], [5] and *Mycobacterium smegmatis* [6]. The degradation of aflatoxin in *M. smegmatis* has been shown to involve two families of F_420_H_2_-dependent reductases [6], but the role of these enzymes in the other species is unknown. The five enzymes characterized from one of these families (the FDR-A family) have 10 to 100 fold more activity for the aflatoxins than do the seven characterized from the other (FDR-B) family [6].

F_420_ is a naturally occurring deazaflavin derivative found in methanogenic *Archaea*, the *Actinomycetales* and some *Proteobacteria* [7]. It is a flavin analog (synthesized from a riboflavin intermediate) that differs from other flavin-derived cofactors, such as flavin mononucleotide (FMN), in having a negatively charged phospholacto γ-glutamyl chain as opposed to a phosphate attached to its ribitol group. The phospholacto γ-glutamyl chain may prevent the cofactor from diffusing across the cell membrane [8], [9]. Despite its structural similarity to FMN, F_420_ is chemically more akin to NAD(P)H as a cofactor in that it only takes part in hydride transfers, and not other oxidative reactions as FMN does. F_420_ also has a redox potential of −350 mV [10], [11], which is slightly more negative than NAD(P)H (−320 mV) [12], and much more so than FMN (−230 mV) [13].

F_420_ is reduced by F_420_-dependent glucose 6-phosphate dehydrogenase (FGD) as the first step in the pentose phosphate pathway in a number of species of *Mycobacteria* and *Nocardia* [7]. In other species, such as *Rhodococcus* and *Nocardioides*, F_420_ is reduced by F_420_:NADPH oxidoreductase [14], [15]. Reduced F_420_ has been implicated in functions as diverse as NO reduction [16], denitration of picric acid [14], [17], malachite green decolorisation [18], antibiotic biosynthesis [19], [20] and PA-824 antituberculosis drug activation [21], [22], [23], as well as aflatoxin degradation [6]. The unique chemistry and low reduction potential of F_420_ could enable it to reduce many of these otherwise relatively recalcitrant molecules.

Recent annotations of sequenced *Actinomycetales* genomes have identified three families of potential F_420_-dependent enzymes [24], two of which involve the FDR-As and FDR-Bs that are able to reduce aflatoxins. These two families are structurally and phylogenetically related to the FMN-dependent pyridoxamine 5′-phosphate oxidase (PNPOx) family. The FDR-A family also includes the deazaflavin-dependent nitroreductase (Ddn) Rv3547, from *Mycobacterium tuberculosis*, that has been shown to reduce and activate the anti-tuberculosis drug PA-824 [23]. There is no experimental evidence as to whether any other enzyme in this family can act as a nitroreductases. The FDR-B enzymes are more closely related to the PNPOx enzymes, but none of the FDR-Bs tested was able to catalyze the oxidation of pyridoxamine 5′-phosphate to pyridoxal phosphate (vitamin B6) in the presence of FMN [6]. The third family containing F_420_-dependent enzymes are TIM barrel proteins that are phylogenetically related to the lumazine synthase enzymes [24]. The functionally described F_420_-dependent members of this class include FGD [24], [25] and enzymes involved in antibiotic biosynthesis [19], [20].

To determine how widespread aflatoxin degrading activity is within the *Actinomycetales* we have cloned and expressed ten FDR-A enzymes from various species across four suborders within the *Actinomycetales*. We have further characterized these enzymes with other furanocoumarins, specifically angelicin, imperatorin and 8-methoxypsoralen (8-MOP) plus khellin, all of which are defence compounds exuded by plant roots that are structurally related to aflatoxins as well as some other structurally related compounds. Some of these molecules also proved to be substrates for some of the FDR-As. Mass spectrometry was carried out to identify reaction products.

## Materials and Methods

### Chemicals

Aflatoxins B1 and G1 were obtained from Sigma-Aldrich (Australia) and Fermentek (Israel), at greater than 98% purity. Angelicin, imperatorin, coumarin, 8-methoxypsoralen, hydroprene, khellin and glucose-6-phosphate were obtained from Sigma-Aldrich, at greater than 98% purity. F_420_ was prepared from soluble fractions of *M. smegmatis* extracts, as per the methods of Isabelle *et al.* [8].

### Cloning, expression and purification of recombinant proteins

Ten previously uncharacterized *M. smegmatis fdr-A* genes were amplified from genomic DNA of strain mc^2^155 using Platinum high fidelity *Taq* polymerase (Invitrogen, USA) and the primer sequences shown in Table S1. One previously characterized *fdr-A*, MSMEG_5998, was also re-isolated as a truncated gene (because it had not expressed well originally and sequence analysis suggested that the 27 N-terminal residues of the encoded protein may be superfluous). The amplicons corresponding to all these genes were recombined into the destination vector pDEST17 via the pDONR201 plasmid of the Gateway System (Invitrogen). Genes for ten other FDR-A enzymes chosen from nine different strains within the *Actinomycetales* (*Frankia alni* ACN14a, *Janibacter sp* HTCC2649, *Marine actinobacterium* PHSC20C1, *Mycobacterium tuberculosis* H37Rv, *Mycobacterium vanbaalenii* PYR-1, *Nocardia farcinica* IFM 10152, *Rhodococcus erythropolis* PR4 (two genes), *Rhodococcus jostii* RHA1, *Streptomyces coelicolor* A3(2)) were synthesized commercially (GeneArt, Germany) in *E. coli-*codon-optimized form and confirmed by sequencing (Micromon, Australia). The ten genes were then recombined into the Gateway destination vector pDEST17. The protein accession numbers for the FDR-A and related enzymes studied here, and their sources, are shown in Table S2.

Expression and purification of the enzymes encoded by these newly amplified or synthesized genes, and re-expression/purification of other FDR-As and FDR-Bs of Taylor *et al.* [6], were carried out following the methods of those authors. Briefly, plasmid DNA was obtained *via* the QiaQuick mini-prep kit (Qiagen) and transformed into *E. coli* BL21 AI cells (Invitrogen). The cells were grown in Luria-Bertani medium containing 100 µg/ml ampicillin for 2 hours at 37°C (when the OD reached about 0.6) at which point they were induced with 0.2% L-arabinose (Sigma-Aldrich) at 28°C for 2 hours. The cells were centrifuged and the cell pellet resuspended in lysis buffer (50 mM NaH_2_PO_4_, 300 mM NaCl, 20 mM imidazole, pH 8.0). The cells were then lysed with an EmulsiFlex-C3 homogeniser (ATA Scientific, Australia) and the recombinant proteins were purified from the soluble cell extract by nickel affinity chromatography on a 0.5 ml bed-volume Ni-NTA resin (Invitrogen). Bound proteins were eluted with increasing concentrations of 250–500 mM imidazole. The purity of the proteins was observed on NuPAGE® Novex® 10% Bis-Tris gels (Invitrogen, Australia) run at 120 V and stained with Coomassie Brilliant Blue. The purified proteins were dialyzed and stored at 4°C in 50 mM NaH_2_PO_4_, pH 8.0. The concentrations of the purified proteins were determined by measuring absorbance at 280 nm using a NanoDrop Spectrophotometer ND1000 (Thermo Fisher Scientific, Australia), with the extinction coefficient of each protein (Table S2) estimated using Vector NTI (Invitrogen).

### Enzyme activity assays

Expressed enzymes were tested for activity against aflatoxin G1 (AFG1) and aflatoxin B1 (AFB1) in the presence of F_420_H_2_ as per the methods of Taylor *et al.* [6]. The assays were carried out in 20 or 200 µl volumes at 22°C with a reaction mix consisting of 0.1–1 µM enzyme, 10 µM F_420_, 2.5 mM glucose-6-phosphate, 0.45 µM FGD, 50 mM Tris HCl, pH 7.5, and 100 µM aflatoxin. Assays with other potential substrates were set up in the same way except that substrate concentrations were adjusted to suit their aqueous solubility; imperatorin was added at 148 µM, 8-MOP at 58 µM, angelicin at 135 µM, coumarin at 100 µM, khellin at 190 µM and hydroprene at 90 µM. Time course high performance liquid chromatography (HPLC) assays were conducted by sampling 5 µl of the reaction mix at defined intervals (2 min–30 min depending on the reaction), using an Agilent 1200 HPLC autosampler, and injecting onto an Agilent Zorbax Eclipse XDB-C18 column (3.5 µm, 2.1×30 mm). The aflatoxins were separated as per the methods of Taylor *et al.* [6]. Coumarin, angelicin, 8-MOP and khellin were separated isocratically with 30% acetonitrile and 0.5% acetic acid in water (v/v). Imperatorin and hydroprene were separated isocratically with 50% acetonitrile and 0.5% acetic acid. Substrates were monitored at 365 nm for aflatoxin, 325 nm for coumarin, 254 nm for imperatorin, 8-MOP, angelicin and khellin, and 265 nm for hydroprene. Specific activities for the substrates were determined by quantifying the loss of substrate using Chemstation software (Agilent). Correlations between activities on different substrates were assessed using Sigma Plot 11.0 (Systat Software, USA).

Reaction products for the furanocoumarins were verified by liquid chromatography mass spectrometry (LCMS) analysis to confirm if the reaction chemistry was as seen with aflatoxins as per previously published methods [6]. Briefly, samples were separated on an Agilent Zorbax XDB-C18 column using an Agilent 1100 Series Binary LC. Accurate mass was determined using an Agilent Time of Flight (ToF) Mass Spectrometer. The results were analyzed using the Analyst QS software (Agilent).

### Phylogenetic methods

FDR-A enzymes (TIGR00026/PF04075/DUF385) within the *Actinomycetales* were identified by searching the NCBI databases on 17/08/2010. 164 FDR-A amino acid sequences (details in Fig. S1) were recovered from twenty different *Actinomycetales* strains (*M. smegmatis* and the nine strains from which new FDR-As were cloned and expressed above, plus *Frankia sp.* EAN1pec, *Kineococcus radiotolerans*, *Mycobacterium avium* 104, *Mycobacterium bovis* AF2122/97, *Mycobacterium gilvum* PYR-GCK, *Mycobacterium sp.* JLS, *Mycobacterium leprae*, *Mycobacterium ulcerans* Agy99 and *Saccharopolyspora erythraea* NRRL 2338 and *Streptomyces galilaeus*). 75 FDR-B and PNPOx enzymes (TIGR00026/PF01243) (from *E. coli*, *Frankia alni* ACN14a, *Homo sapiens*, *Mycobacterium* sp. JLS, *M. smegmatis*, *M. tuberculosis* H37Ra and *M. vanbaalenii* PYR-1), recovered by similar methods, were used as outgroups. Amino acid sequences were aligned by ClustalW and a phylogenetic tree constructed from full-length sequences using the Minimum Evolution method as implemented in MEGA 4.0 [26], with pairwise deletion, Poisson correction and 1000 bootstrap replicates.

## Results

### Orthologues of FDR-A enzymes from numerous *Actinomycetales* reduce aflatoxin

Genes encoding ten FDR-A enzymes from nine different *Actinomycetales* were synthesized, cloned and expressed (Fig. S2) to determine how widespread the FDR-catalyzed reduction of aflatoxins might be within this order. Four of the strains (*N. farcinica*, *R. jostii*, *S. coelicolor* A3(2) and the nitrogen fixing plant symbiont *F. alni*) were isolated from soil and therefore might encounter aflatoxins, but the remainder were from environments far less likely to contain aflatoxins (*M. vanbaalenii* is from estuarine sediments; *Janibacter* sp. HTCC2649, marine *Actinobacterium* and *R. erythropolis* PR4 are from marine environments; and *M. tuberculosis* H37Rv is a laboratory strain of a human pathogen). The ten enzymes (Table S2) were selected because of their close sequence similarity to one or other of the three previously characterized FDR-A enzymes, MSMEG_3356, _2850 and _5998 from *M. smegmatis*, with the greatest activities against AFG1 (≳ 10 µmol min^−1^ µmol^−1^ enzyme) [6]. Qualitative analysis of substrates AFG1 and AFB1 after overnight incubation with the enzymes and F_420_H_2_ revealed that nine of the enzymes could reduce both aflatoxins, the exception being the SCO7200 enzyme from the soil bacterium *S. coelicolor* A3(2), which was unable to reduce either (data not shown).

### FDR-A enzymes fall into two functionally distinct clades

**Figure 1:**
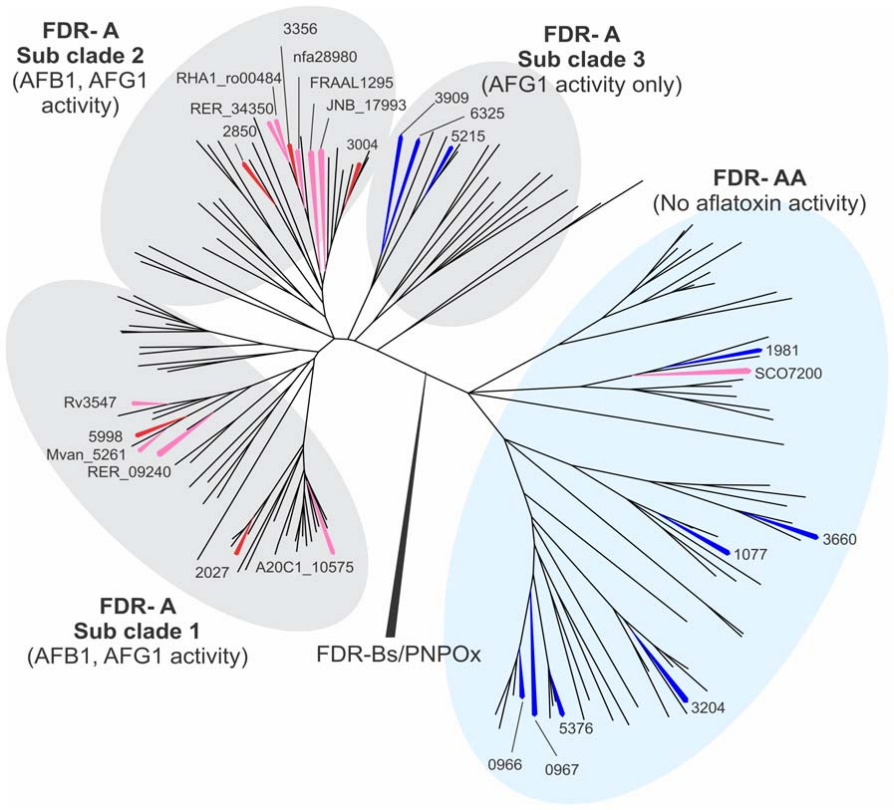
Phylogeny of 164 FDR-A enzymes from 20 *Actinomycetales* genomes. Phylogenetic methods and the genomes used are given in the Materials and Methods. For clarity, only the FDR-As that have been characterized biochemically are labeled (*M. smegmatis* previously characterized (red), *M. smegmatis* characterized herein (blue) and the other *Actinomycetales* species characterized herein (pink)). The *M. smegmatis* enzymes are labeled with their locus tag numbers (the MSMEG_ prefix being deleted for clarity) while the enzymes from the other species are labeled with their complete locus tag. All the characterized enzymes are also given simple number designations (in brackets) to aid comparison with later figures. A fully labeled version of this phylogeny is given as Figure S1.

To understand the distribution of the active and inactive FDR-A enzymes from the various *Actinomycetales* tested above, we constructed a phylogenetic tree of 164 FDR-As from twenty sequenced *Actinomycetales* genomes (including *M. smegmatis* and the nine species selected above; Fig. S1). The number of FDR-As identified in these genomes ranged from one in *M. leprae* up to 15 in *Mycobacterium* species JLS and *M. smegmatis*. This tree (Fig. 1), which we rooted with the sequences of PNPOx and FDR-B enzymes, revealed two major clades of FDR-As, one of which contained all 19 of the enzymes shown above and by Taylor *et al.* [6] to have activity against either or both of AFG1 and AFB1 (and for which we retain the name FDR-A), and the other of which contained the SCO7200 enzyme above from *S. coelicolor* that lacked activity against either (and which we rename FDR-AA). All 20 genomes had at least one representative in each clade (apart from *M. leprae* which has only one FDR-AA enzyme), although there was considerable unevenness in the distribution of species' representatives across subclades (see also Fig. S1).

The phylogeny also showed three distinct subclades within the FDR-A clade, with all the previously characterized *M. smegmatis* enzymes within subclades 1 and 2. The third subclade contained three previously uncharacterized *M. smegmatis* enzymes (MSMEG_3909, _5215 and _6325) (Fig. 1). We therefore cloned and expressed these three enzymes, plus seven *M. smegmatis* FDR-AA enzymes and assayed their activities against AFB1 and AFG1. Qualitative analysis after overnight incubations showed that the seven additional FDR-AA enzymes could not reduce AFG1 or AFB1 while the three FDR-A3 enzymes were all active against AFG1, albeit not AFB1. These results suggest that some aflatoxin reduction activity is generally present in the FDR-A clade and generally absent in the FDR-AA clade, and that the activity in the third subclade of the FDR-As does not extend to AFB1.

### FDR enzymes reduce furanocoumarins

All 17 FDR-A and eight FDR-AA enzymes expressed and purified in this study, along with two *M. smegmatis* FDR-B enzymes, MSMEG_3380 and MSMEG_6848, from Taylor *et al*. [6], were assayed quantitatively for activity against AFG1 and AFB1 and six other potential substrates; the α,β-unsaturated esters angelicin, imperatorin, 8-MOP, coumarin and hydroprene, plus the α,β-unsaturated ketone, khellin.

**Figure 2:**
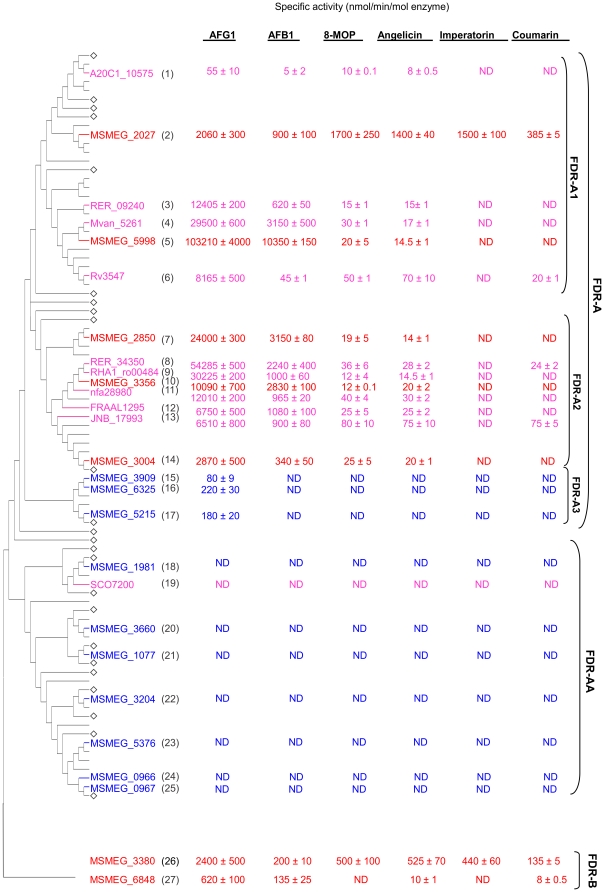
Specific activities of heterologously expressed and purified FDR-A and -B enzymes against different substrates. *M. smegmatis* enzymes characterized previously (red) or herein (blue) and enzymes from the other *Actinomycetales* species characterized herein (pink) are color-coded and labeled as per Fig. 1 and their phylogenetic relationships are shown on the left (reformatted from Fig. 1). Subclades that have been condensed are labeled ◊. Specific activities are given as means and standard errors of at least three biological replicates. Substrates were tested with 1 µM enzyme except for MSMEG_5998 (0.1 µM) for AFG1 and AFB1 and MSMEG_2027 (0.5 µM) for imperatorin. ND – Not detected.

**Figure 3:**
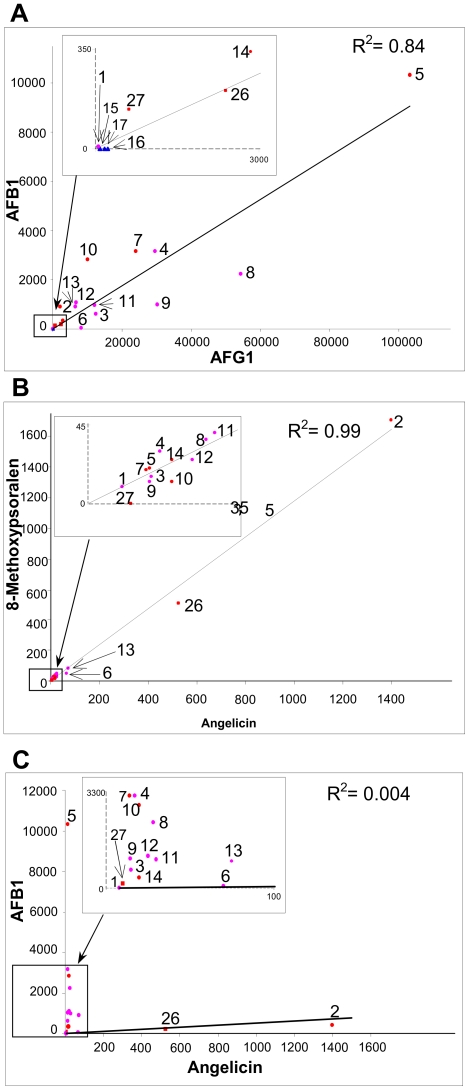
Pairwise correlations among the AFB1, AFG1, angelicin and 8-methoxypsoralen activities of FDR-A1/2/3 and FDR-B enzymes. The R^2^ values for each pair of activities are given on each graph. Enzymes are numbered as per Fig. 2.

Values for specific activities with AFB1 and AFG1 for the FDR-A enzymes from the other *Actinomycetales* are broadly comparable to those of the previously characterized *M. smegmatis* FDR-As (Fig. 2). There is again a strong trend for higher activities with AFG1 than AFB1, although overall there is a strong positive correlation between the two activities (Fig. 3). The most active enzyme for both substrates remains MSMEG_5998 (103±4 and 10.3±0.2 µmol min^−1^ µmol^−1^ enzyme for AFG1 and AFB1, respectively). The next highest activity for AFB1 was for the *M. vanbaalenii* enzyme, which sits in the same subclade (FDR-A1) as MSMEG_5998, Mvan_5261 (3.1±0.6 µmol min^−1^ µmol^−1^ enzyme) and the *M. smegmatis* enzymes MSMEG_2850 and MSMEG_3356 (3.1 and 2.8 µmol/min/µmol enzyme, respectively). The next three highest values for AFG1 activity were from other species: *R. erythropolis*, RER_34350; *R. jostii*, RHA1_ro00484 and *M. vanbaalenii*, Mvan_5261 (54.3±0.5, 30.2±0.2 and 29.5±0.6 µmol min^−1^ µmol^−1^ enzyme, respectively), although only one of the latter is from a soil organism (*R. jostii*). The subclades FDR-A1 and -A2 containing MSMEG_5998 and MSMEG_3356 contain all of the higher activity enzymes but there is also considerable variation within these subclades with regards to their aflatoxin reduction capability.

All of the FDR-A1 and FDR-A2 enzymes across the different species which were able to reduce both AFG1 and AFB1 were also able to reduce angelicin and 8-MOP, whereas no activity for these two furanocoumarins was observed for the three FDR-A3s that could only reduce AFG1 or for any of the FDR-AA enzymes. Interestingly, the two FDR-Bs could reduce angelicin and one could also reduce 8-MOP. Specific activities for the two furanocoumarins were strongly correlated with each other (r^2^ = 0.99) but bore little relationship to aflatoxin activities (r^2^<0.01) (Fig. 3).

With the exception of one FDR-A enzyme, MSMEG_2027, and the FDR-B, MSMEG_3380, activities against angelicin and 8-MOP were quite low. These two enzymes were also the only ones from *M. smegmatis* that had any activity against coumarin and imperatorin (Fig. 2). In fact, their activity for the furanocoumarins was approximately 2 fold greater than their AFB1 activity (in contrast to the other FDR-A enzymes which had at least 10 fold more activity for AFB1 than the furanocoumarins). This showed that some FDR enzyme activities have diverged so as to utilise different α-β unsaturated esters. Thus, most of the FDR-A1, -A2 and a couple of FDR-B enzymes are capable of significant substrate promiscuity.

No activity with hydroprene and khellin was seen for any of the FDR enzymes. In the case of khellin this was probably because it is an α,β-unsaturated ketone rather than an ester and its α,β-unsaturated bond is stabilized by a methyl group. Hydroprene is a linear molecule that does have an α,β-unsaturated ester moiety but its double bond is also stabilized by a methyl group at the β-carbon.

**Figure 4:**
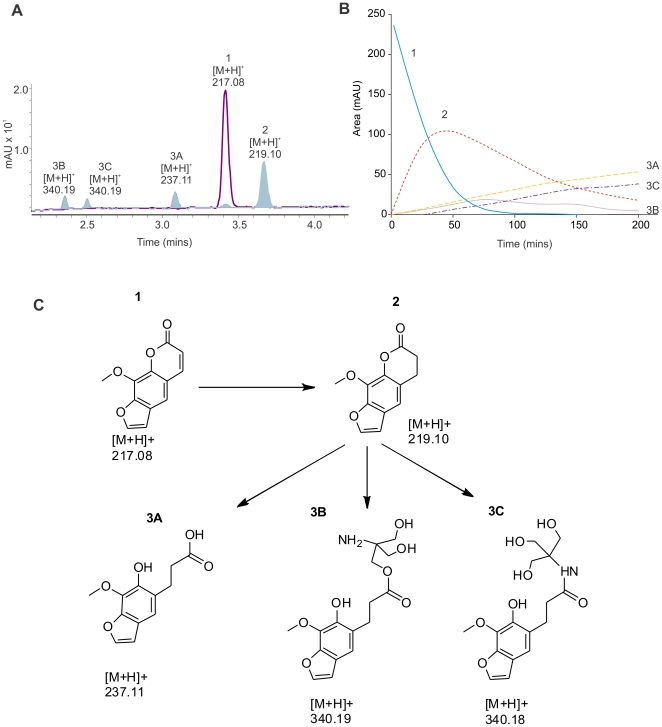
Reduction and spontaneous hydrolysis of 8-MOP. 8-MOP was reduced with MSMEG_2027 and analysed by LCMS. Spectra from both diode array (A) and MS ToF were recorded, diode array peaks are labeled with [M+H]^+^. The reaction was monitored every 20 minutes and the area of each reaction product was quantified. Predicted reaction products for 8-MOP and its spontaneous hydrolysis products are shown (C).

Unlike aflatoxin [6], the reduced furanocoumarins yielded detectable breakdown products, as measured by the absorbance at 254 nm. The presence of these products was quantified over a 12 hour time course taking measurements every 20 minutes; the HPLC trace and time course for 8-MOP are shown in Fig. 4 A and B, respectively. In all cases the substrate (1) is reduced to an unstable dihydro reaction product (Fig. 4C, 2) as confirmed by the increase in molecular weight of 2.02 m/z, as measured by LCMS ToF. The dihydro-furanocoumarin was then spontaneously hydrolyzed into three secondary products (3A–3C), all appearing after the detection of the initial reduction product. The first of the secondary reaction products (3A) corresponds to a ring opening hydrolysis as previously characterized by a similar reduction by XenA [27]. However, the other two products (3B and 3C) were observed with an m/z ratio of 121.08 greater than the initial reaction product. This corresponds to the condensation of Tris buffer with the unstable dihydro-furanocoumarin reaction product. The dihydro-furanocoumarin product may react with Tris by two methods, which may account for the two observed product peaks. Firstly, it may be ring opened by any one of the three hydroxyl groups of Tris by alcoholytic ring opening [28]. Secondly, the carbon associated with the ester functional group may be attacked by the amine via nucleophilic substitution. Further experiments are required to confirm these reaction products by NMR.

## Discussion

In previous work we discovered five FDR-A enzymes from *M. smegmatis* which could all reduce aflatoxins [6]. We have now tested another ten FDR-A enzymes from various other *Actinomycetales* from a wide range of ecological niches and found that nine of them can also reduce both the aflatoxins (AFB1 and AFG1) that we have assayed. This suggests that aflatoxin reducing activity is widespread in this family of enzymes and, as the family of enzymes is itself widespread in the *Actinomycetales*, it suggests that a capacity to degrade aflatoxins could be widespread in this order. This is consistent with previous reports of aflatoxin degradation in other species of *Actinomycetales*, such as *N. corynebacteriodes* [2], *R. erythropolis*, and *M. fluoranthenivorans* sp. Nov DSM44556^T^ [3], [4], [5].

Phylogenetic analyses showed that the one *Actinomycetales* FDR-A enzyme (SCO7200) we tested that was unable to reduce either AFB1 or AFG1 was only distantly related to all the aflatoxin-active enzymes. Functional analysis of seven comparatively close relatives of SCO7200 from *M. smegmatis* then found that they were also incapable of reducing the aflatoxins. It appears that the clade containing these enzymes, which we denote here as FDR-AA, represents a distinct functional as well as phylogenetic entity. This functional distinction was borne out by our subsequent analysis of the enzymes' activities for various furanocoumarins, which likewise showed that some furanocoumarin activity was common among the FDR-As but lacking in the FDR-AAs tested.

There are two other lines of evidence in our data showing functional evolution within the FDR-A/FDR-AA phylogeny. Firstly, we found a small subclade of FDR-As, the FDR-A3s, that could reduce AFG1 but not AFB1. Secondly, the capability of reducing at least some of the furanocoumarins proved to be widespread among the other FDR-As and absent from the three FDR-A3s and eight FDR-AAs tested. The functions of the FDR-AAs thus remain unknown. In fact, to the best of our knowledge there is no empirical functional data for any of the FDR-AA enzymes that we could find in our database searches.

Indeed we have no direct evidence that the FDR-AA clade even uses F_420_. Given their relationship to the highly conserved FMN-utilizing PNPOx enzymes [6], it is possible that the FDR-AA enzymes may have evolved to use another flavin cofactor, perhaps FMN or maybe even another *Actinomycetales*-specific flavin analogue such as roseoflavin [29], [30], [31]. It is notable that the structural and amino-acid sequence identity of the FDR-A and -AA enzymes (Fig. S3) shows that the putative phosphate binding residue W76 is highly conserved throughout both classes, suggesting a conserved need for the cofactor to contain a ribitol phosphate chain. Furthermore, all of the other residues identified in the active site are conserved in both FDR-A and -AA, with the notable exception of Y124, which is only highly conserved in the active FDR-A enzymes but not in the -AA enzymes. Further mutagenesis and functional assays are required to identify the role of these residues in the FDR enzymes and provide insight into the functional evolution of this family.

While the FDR-As are generally able to reduce the aflatoxins and some of them can also reduce coumarin and certain furanocoumarins, there are large, quantitative differences in the activities of the different FDR-As for these compounds. This activity variation is highly correlated for the two aflatoxins and also for the two furanocoumarins angelicin and 8-MOP, but there is very little correlation between activities for the aflatoxin and the two furanocoumarin substrates. The aflatoxins and (furano)coumarins in this study may represent only a small proportion of the possible substrates for these enzymes because the α,β-unsaturated lactone moiety that appears to be required for activity occurs in a wide variety of xenobiotics and antibiotics. Such compounds may include UV oxidised polyaromatic hydrocarbons [32], plant derived lactones such as digitalis [33] and other antibiotics including leptomycin [34]. It remains to be determined as to whether the aflatoxins or (furano)coumarins are in fact physiological substrates for any of the enzymes here; some of the host organisms have ecologies which would not likely encompass these specific compounds.

Once the (furano)coumarins are reduced by the FDR-As they are likely to be further catabolized in the *Actinomycetales* through the catechol catabolic pathway, providing substrates for the citric acid cycle [35]. In the case of coumarin, dihydrocoumarin resulting from the FDR-catalyzed reduction reaction would be spontaneously hydrolyzed to 3-hydroxyphenyl propionic acid, which could be further hydrolyzed to 2,3-dihydroxyphenyl propionic acid by melilotate hydroxylase [36], enabling ring meta-cleavage by extradiol dioxygenases [37], [38]. After cleavage, the resulting succinate would enter the citric acid cycle. The other furanocoumarin compounds are similarly reduced by the FDRs and after further hydrolysis should be ring cleaved by extradiol dioxygenases and lysed to produce succinate, thus also providing a carbon source. Other metabolic fates might also be possible for the possible hydrolyzed intermediates, such as through condensation by 4-coumarate-CoA ligase [39]. Further experiments are required to confirm the hydrolyzed ring products of dihydrocoumarin, and the metabolic fate of coumarin and its furano derivatives in the F_420_ containing *Actinomycetales*.

To gain further insight into the potential substrates for the FDR enzymes we interrogated Biocyc (http://biocyc.org/comp-genomics) for potential operons with at least partly conserved gene complements in which the genes encoding the *M. smegmatis* FDR-A/AA/B enzymes might sit (Table S3). Only one gene, encoding the FDR-A1 enzyme MSMEG_5998, was predicted to be in such an operon. One of the other two genes in this operon, encoding the enzyme MSMEG_5997, was found to be conserved in some related species but is not annotated with any putative function or structural domain. The other, encoding MSMEG_5996, is predicted to be an acetyl-CoA acetyl transferase gene. Further analysis using NCBI gene view and BLAST revealed that regions in the immediate vicinity of genes encoding *M. vanballenii*, *R. erthropolis* and *M. tuberculosis* homologues of MSMEG_5998 also contain acetyl-CoA acetyl transferase genes (Table S3). Acetyl-CoA acetyl transferases are known to perform a broad range of functions in central metabolism and biosynthesis [40]. They are also involved in *Actinomycete*- specific pathways such as mycothiol formation [41]. Genes encoding FDR-As in *Streptomyces* species have been found in antibiotic biosynthetic operons [42], [43], [44], although their exact role in the biosynthesis has not been determined. No operons with at least partially conserved gene complements were found for the genes encoding enzymes in the FDR subclades -A2, -A3 or in the FDR-AA and -B clades.

F_420_ has been shown to be a non-essential cofactor in *M. smegmatis* [6], [18] but this and other studies have shown that F_420_ is required for the metabolism of several otherwise recalcitrant molecules. As noted above, the aflatoxins and plant furanocoumarins studied here may only be model substrates for most of the FDR-A enzymes, many of which are in organisms found in environments not likely to encounter such compounds. The FDR-As are a divergent family with few conserved amino acids, thus suggesting the ability to adapt to new substrates and perhaps new cofactors. We have proposed a role of these enzymes in the secondary metabolism of toxic compounds, including antibiotic biosynthesis. We further suggest that these enzymes, although diverse, may be important to the F_420_-containing *Actinomycetales* to adapt to the harsh environments in which they can be found, aiding in the catabolism of many otherwise recalcitrant molecules.

## Supporting Information

Figure S1 **Phylogenetic tree of FDR-A enzymes used in this study.**
(TIF)

Figure S2 **SDS-PAGE of his-tagged FDR-A enzymes from ten different** ***Actinomycetales*** **species.** Whole cell (WC) and soluble protein (S) fractions of each protein were recombinantly expressed in *E. coli* BL21-AI cells and separated on a 10% SDS-PAGE with a Biorad (Australia) Precision Plus Protein Standards molecular weight marker (M). *E. coli* BL 21- AI cells were used as control (control), The expressed proteins were: FRAAL1295 (1), SCO7200 (2), A20C1_10575 (3), RER_09240 (4), nfa28980 (5), JNB_17993 (6), RER_34350 (7), RHA1_ro00484 (8), Mvan_5261 (9) and Rv3547 (10).
(TIF)

Figure S3 **Comparison of putative deazaflavin pocket and glutamate chain binding sites in the FDR-As.** Amino acid substitutions within the putative deazaflavin binding pocket as well as the γ-glutamate chain are shown in comparison to the crystal structure of MSMEG_3356. Conserved amino acids compared with MSMEG_3356 are shown with an * (in red). FDR-A enzymes showing specific activities above 10,000 nmol/min/µmol enzyme are highlighted with a grey background. FDR-A enzymes are arranged in the same sequence as in Figure 2.
(TIF)

Table S1 **Primers used for protein expression work.**
(DOCX)

Table S2 **Genes expressed in this study.**
(DOCX)

Table S3 **Genes observed in the same reading frame as FDR genes.**
(DOCX)
