# Supplementary figures and images for: F420H2-Dependent Degradation of Aflatoxin and other Furanocoumarins Is Widespread throughout the Actinomycetales
Source: PLoS One. 2012 Feb 27;7(2):e30114. doi: 10.1371/journal.pone.0030114 (PMC3288000; doi:10.1371/journal.pone.0030114)

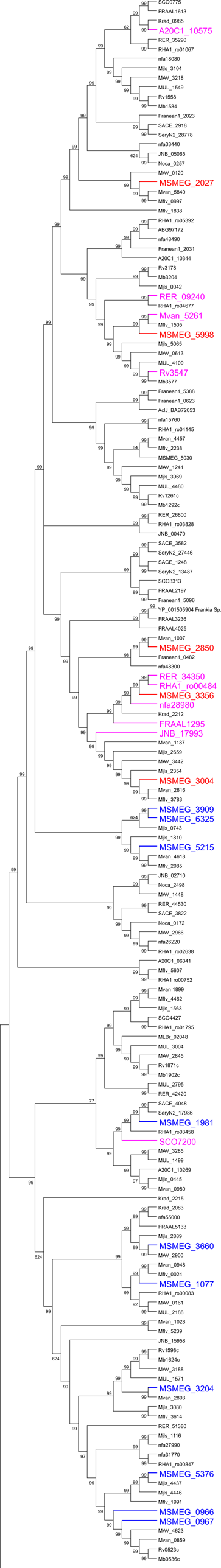

Supplement: Figure S1 — Phylogenetic tree of FDR-A enzymes used in this study. (TIF) [file pone.0030114.s001.tif]

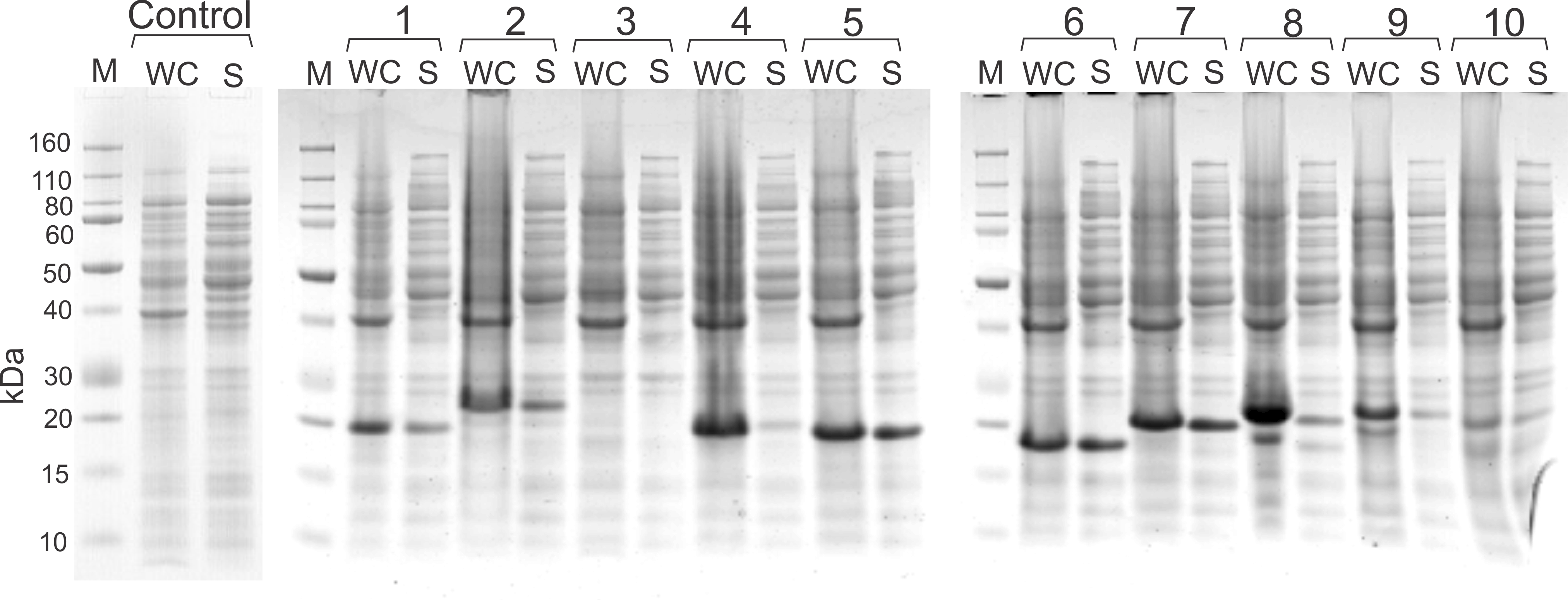

Supplement: Figure S2 — SDS-PAGE of his-tagged FDR-A enzymes from ten different Actinomycetales species. Whole cell (WC) and soluble protein (S) fractions of each protein were recombinantly expressed in E. coli BL21-AI cells and separated on a 10% SDS-PAGE with a Biorad (Australia) Precision Plus Protein Standards molecular weight marker (M). E. coli BL 21- AI cells were used as control (control), The expressed proteins were: FRAAL1295 (1), SCO7200 (2), A20C1_10575 (3), RER_09240 (4), nfa28980 (5), JNB_17993 (6), RER_34350 (7), RHA1_ro00484 (8), Mvan_5261 (9) and Rv3547 (10). (TIF) [file pone.0030114.s002.tif]

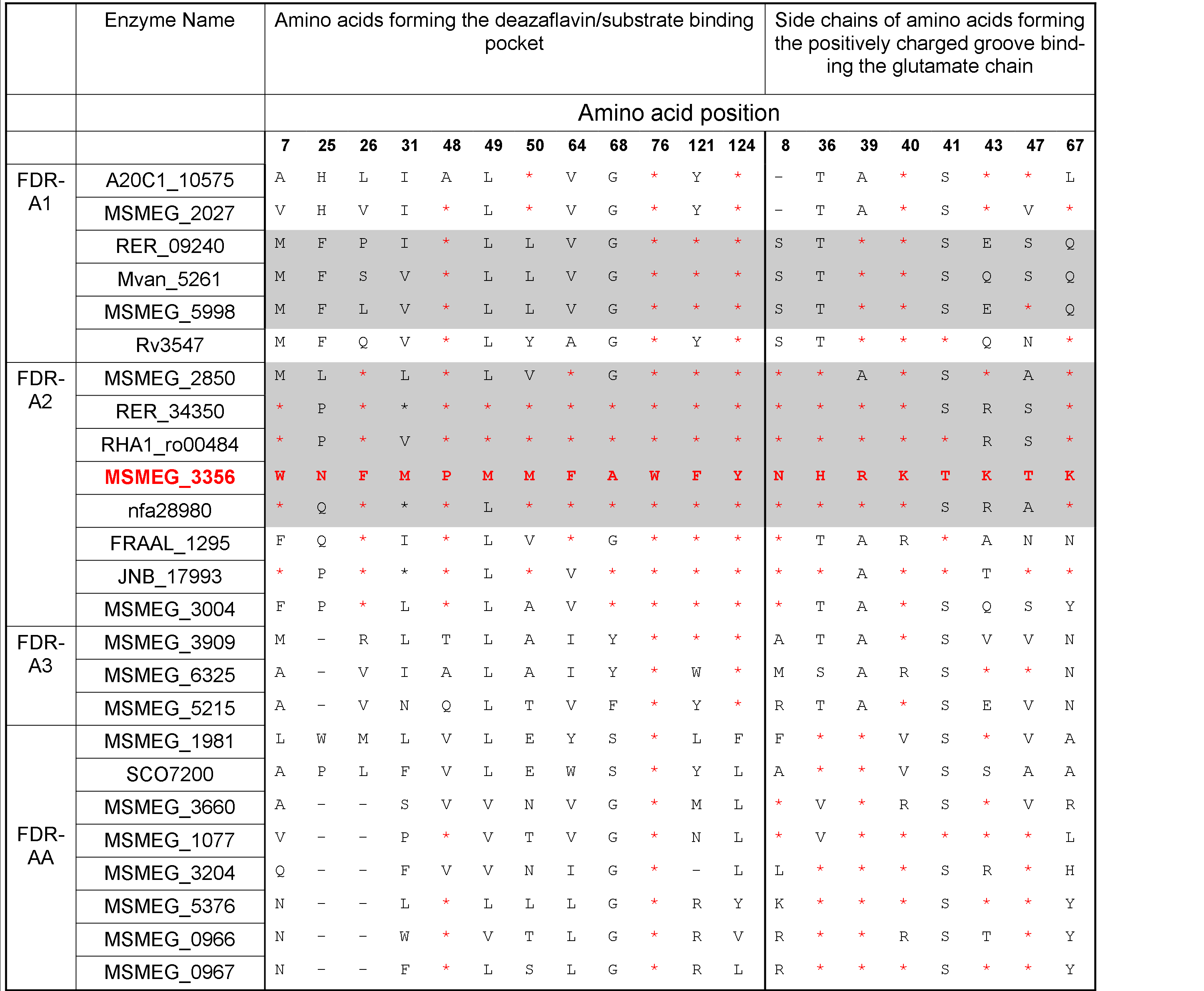

Supplement: Figure S3 — Comparison of putative deazaflavin pocket and glutamate chain binding sites in the FDR-As. Amino acid substitutions within the putative deazaflavin binding pocket as well as the γ-glutamate chain are shown in comparison to the crystal structure of MSMEG_3356. Conserved amino acids compared with MSMEG_3356 are shown with an * (in red). FDR-A enzymes showing specific activities above 10,000 nmol/min/µmol enzyme are highlighted with a grey background. FDR-A enzymes are arranged in the same sequence as in Figure 2. (TIF) [file pone.0030114.s003.tif]
