# Supplementary material for: F420H2-Dependent Degradation of Aflatoxin and other Furanocoumarins Is Widespread throughout the Actinomycetales
Source: PLoS One. 2012 Feb 27;7(2):e30114. doi: 10.1371/journal.pone.0030114 (PMC3288000; doi:10.1371/journal.pone.0030114)
Supplement: Table S1 — Primers used for protein expression work. (DOCX) [file pone.0030114.s004.docx]

**Table S1. Primers used for protein expression work**

| **Primer name** | **Primer sequence** |
| --- | --- |
| MSMEG_0966 AttB1 | GGGGACAAGTTTGTACAAAAAAGCAGGCTTAATGCTGTTGGCCGACGAGCGCG |
| MSMEG_0966 AttB2 | GGGGACCACTTTGTACAAGAAAGCTGGGTATCAGTCCGTCCGCCGCAA |
| MSMEG_0967 AttB1 | GGGGACAAGTTTGTACAAAAAAGCAGGCTTAATGCAACTTCCGCAATGG |
| MSMEG_0967 AttB2 | GGGGACCACTTTGTACAAGAAAGCTGGGTACTAGACCCGACGAAGCAG |
| MSMEG_1077 AttB1 | GGGGACAAGTTTGTACAAAAAAGCAGGCTTAATGCGCACCGAACGTATC |
| MSMEG_1077 AttB2 | GGGGACCACTTTGTACAAGAAAGCTGGGTATCAGAGCGGGTCGAGACG |
| MSMEG_1981 AttB1 | GGGGACAAGTTTGTACAAAAAAGCAGGCTTAATGAAGATCACCAAGAGGGTC |
| MSMEG_1981 AttB2 | GGGGACCACTTTGTACAAGAAAGCTGGGTATCATCCCGGTGTCCGAGG |
| MSMEG_3204 AttB1 | GGGGACAAGTTTGTACAAAAAAGCAGGCTTAATGCTGTTCCCGCCCTGG |
| MSMEG_3204 AttB2 | GGGGACCACTTTGTACAAGAAAGCTGGGTATCAAACGATTTCGGCTAC |
| MSMEG_3660 AttB1 | GGGGACAAGTTTGTACAAAAAAGCAGGCTTAATGACCAGGTATGACGAACC |
| MSMEG_3660 AttB2 | GGGGACCACTTTGTACAAGAAAGCTGGGTATCAGCTCGTCTCGACGAG |
| MSMEG_3909 AttB1 | GGGGACAAGTTTGTACAAAAAAGCAGGCTTAATGGCCGACCTGTTGAAC |
| MSMEG_3909 AttB2 | GGGGACCACTTTGTACAAGAAAGCTGGGTATCACTGACGCTCGGGTGT |
| MSMEG_5215 AttB1 | GGGGACAAGTTTGTACAAAAAAGCAGGCTTAATGCCGCTGCCCTACGTGGAT |
| MSMEG_5215 AttB2 | GGGGACCACTTTGTACAAGAAAGCTGGGTATCAGCGCGGCGTGAGGCGGA |
| MSMEG_5376 AttB1 | GGGGACAAGTTTGTACAAAAAAGCAGGCTTAATGCGTGCGCCTCGACAAGTCG |
| MSMEG_5376 AttB2 | GGGGACCACTTTGTACAAGAAAGCTGGGTATCAGCCCGCTGAGCCCCC |
| MSMEG_6325 AttB1 | GGGGACAAGTTTGTACAAAAAAGCAGGCTTAATGGATGACAAGCTCCACG |
| MSMEG_6325 AttB2 | GGGGACCACTTTGTACAAGAAAGCTGGGTATCATGGGTCGGCGGGCGT |
| MSMEG_5998T AttB1 | GGGGACAAGTTTGTACAAAAAAGCAGGCTTAATGTCGCGCTTCCAGACGTTC |
