# Supplementary material for: F420H2-Dependent Degradation of Aflatoxin and other Furanocoumarins Is Widespread throughout the Actinomycetales
Source: PLoS One. 2012 Feb 27;7(2):e30114. doi: 10.1371/journal.pone.0030114 (PMC3288000; doi:10.1371/journal.pone.0030114)
Supplement: Table S2 — Genes expressed in this study. (DOCX) [file pone.0030114.s005.docx]

**Table S2. Genes expressed in this study.**

| **Gene/Plasmid** | **Protein Accession number** | **Molar extinction coefficient (M^−1^ cm^−1^)** | **Source Organism** | **Environmental origin** |
| --- | --- | --- | --- | --- |
| **TIGR Locus tag** |  |  |  |  |
| FRAAL1295 | YP_711546 | 17330 | *F. alni* ACN14a | Plant symbiont - Soil |
| JNB_17993 | ZP_00996801 | 23470 | *Janibacter species* HTCC2649 | Aquatic, Marine |
| SCO7200 | NP_631257 | 27670 | *S. coelicolor* A3(2) | Soil |
| nfa28980 | YP_119109 | 24180 | *N. farcinica* IFM 10152 | Soil |
| Mvan_5261 | YP_956038 | 41370 | *M. vanbaalenii* PYR-1 | PAH contaminated estuarine sediments |
| Rv3547 | NP_218064 | 41370 | *M. tuberculosis* H37Rv | Human pathogen |
| RER_09240 | BAH31632 | 37650 | *R. erythropolis* PR4 1 | Marine |
| RER_34350 | BAH34143 | 25460 | *R. erythropolis* PR4 2 | Marine |
| A20C1_10575 | ZP_01131276 | 34280 | *Marine actinobacterium* PHSC20C1 | Marine |
| RHA1_ro00484 | YP_700478 | 25460 | *R. jostii* RHA1 | Soil |
| **Genes Cloned** |  |  |  |  |
| MSMEG_0966 | YP_885368 | 23470 | *M. smegmatis* mc^2^155 genomic DNA | Soil |
| MSMEG_0967 | YP_885369 | 29160 | *M. smegmatis* mc^2^155 genomic DNA | Soil |
| MSMEG_1077 | YP_885473 | 23590 | *M. smegmatis* mc^2^155 genomic DNA | Soil |
| MSMEG_1981 | YP_886345 | 24750 | *M. smegmatis* mc^2^155 genomic DNA | Soil |
| MSMEG_3204 | YP_887518 | 19060 | *M. smegmatis* mc^2^155 genomic DNA | Soil |
| MSMEG_3660 | YP_887963 | 31720 | *M. smegmatis* mc^2^155 genomic DNA | Soil |
| MSMEG_3909 | YP_888200 | 23140 | *M. smegmatis* mc^2^155 genomic DNA | Soil |
| MSMEG_5215 | YP_889461 | 19890 | *M. smegmatis* mc^2^155 genomic DNA | Soil |
| MSMEG_5376 | YP_889619 | 28710 | *M. smegmatis* mc^2^155 genomic DNA | Soil |
| MSMEG_6325 | YP_890543 | 48340 | *M. smegmatis* mc^2^155 genomic DNA | Soil |
