# Supplementary material for: F420H2-Dependent Degradation of Aflatoxin and other Furanocoumarins Is Widespread throughout the Actinomycetales
Source: PLoS One. 2012 Feb 27;7(2):e30114. doi: 10.1371/journal.pone.0030114 (PMC3288000; doi:10.1371/journal.pone.0030114)
Supplement: Table S3 — Genes observed in the same reading frame as FDR genes. (DOCX) [file pone.0030114.s006.docx]

**Table S3: Genes observed in the same reading frame as FDR genes.**

| **FDR subclade** | **Locus number^1^** | **Chromosome position** | **Annotated function** | **Similarity within the (sub)clade** |
| --- | --- | --- | --- | --- |
| **FDR-A Subclade 1** | **MSMEG_2027** | 2,106,472 - 2,106,894 | **F_420_ dependent reductase** | No similarity to other Subclade 1 enzymes. |
|  | MSMEG_2028 | 2,106,948 – 2,108,135 | Chloramphenicol resistance protein |  |
|  | MSMEG_2029 | 2,108,135 – 2,108,896 | 3-ketoacyl –ACP/CoA  reductase |  |
|  | RER_09220 | 1,016,583 – 1,017,254 | Hypothetical protein | All operons have the acetyl-CoA  acetyltransferase gene |
|  | RER_09230 | 1,017,349 – 1,017,762 | Hypothetical protein |  |
|  | **RER_09240** | 1,017,820 – 1,018,308 | **F_420_ dependent reductase** |  |
|  | RER_09250 | 1,018,310 – 1,019,473 | Acetyl-CoA acetyltransferase |  |
|  | Mvan_5259 | 5,635,313 – 5,636,476 | Acetyl-CoA acetyltransferase |  |
|  | Mvan_5260 | 5,636,487 – 5,636,870 | Hypothetical protein |  |
|  | **Mvan_5261** | 5,636,881 – 5,637,387 | **F_420_ dependent reductase** |  |
|  | Mvan_5262 | 5,637,419 - 5,638,684 | Phosphogycerate mutase |  |
|  | Mvan_5263 | 5,638,789 – 5,639,505 | Putative GAF sensor protein |  |
|  | MSMEG_5996 | 6,062,510 – 6,063,673 | Acetyl- CoA  acetyltransferase |  |
|  | MSMEG_5997 | 6,063,676 – 6,064,056 | Hypothetical protein (74% identity to Mvan_5260) |  |
|  | **MSMEG_5998** | 6,064,064 – 6,064,543 | **F_420_ dependent reductase** |  |
|  | fadA5 | 3,985,557 – 3,986,732 | Acetyl- CoA  acetyltransferase |  |
|  | **Rv3547** | 3,986,844 – 3,987,299 | **F_420_ dependent reductase,**  Nitroreductase (Ddn)  Reference: Manjunatha, 2006. |  |
| **FDR-A Subclade 2** | MSMEG_2849 | 2,913,021 – 2,913,422 | Transcriptional regulatory protein | None |
|  | **MSMEG_2850** | 2,913,508 – 2,913,930 | **F_420_ dependent reductase** |  |
|  | **RHA1_ro00484** | 567,522 – 567,961 | **F_420_ dependent reductase** |  |
|  | RHA1_ro00485 | 568,128 – 568,571 | Hypothetical protein |  |
|  | MSMEG_3353 | 3,428,505 – 3,428,870 | Hypothetical protein |  |
|  | MSMEG_3354 | 3,429,446 – 3,430,054 | TetR family transcriptional regulator |  |
|  | MSMEG_3355 | 3,430,051 – 3,430,938 | Hypothetical protein |  |
|  | **MSMEG_3356** | 3,431,015 – 3,431,353 | **F_420_ dependent reductase** |  |
|  | MSMEG_3357 | 3,431,438 – 3,432,004 | Metal-dependent phosphohydrolase |  |
|  | MSMEG_3358 | 3,432,244 – 3,432,795 | YaeQ protein |  |
|  | MSMEG_3359 | 3,433,116 – 3,433,571 | cis-3- choloroacrylic acid dehalogenase |  |
|  | nfa28970 | 3,080,850 – 3,081,731 | Hypothetical protein |  |
|  | **nfa28980** | 3,081,859 – 3,082,287 | **F_420_ dependent reductase** |  |
|  | FRAAL1294 | 1,396,190 – 1,397,344 | Putative oxidoreductase |  |
|  | **FRAAL1295** | 1,397,391 – 1,397,840 | **F_420_ dependent reductase** |  |
|  | FRAAL1296 | 1,398,028 – 1,398,414 | Putative monooxygenase |  |
|  | MSMEG_3003 | 2,070,138 – 3,071,934 | Aspartyl- tRNA synthetase |  |
|  | **MSMEG_3004** | 3,071,978 – 3,072,433 | **F_420_ dependent reductase** |  |
| **FDR-A Subclade 3** | **MSMEG_3909** | 3,980,700 – 3,981,236 | **F_420_ dependent reductase** | None |
|  | MSMEG_3911 | 3,981,247 – 3,981,558 | Oxidoreductase |  |
|  | MSMEG_3912 | 3,981,603 – 3,982,166 | Acetyl CoA reductase |  |
|  | MSMEG_6323 | 6,391,100 – 6,391,510 | Hypothetical protein |  |
|  | MSMEG_6324 | 6,391,470 – 6,393,257 | Peroxidase |  |
|  | **MSMEG_6325** | 6,393,250 – 6,393,789 | **F_420_ dependent reductase** |  |
|  | MSMEG_5213 | 5,310,853 – 5,311,533 | Hypothetical protein |  |
|  | MSMEG_5214 | 5,311,646 – 5,312,170 | RNA polymerase sigma- 70 factor |  |
|  | **MSMEG_5215** | 5,312,196 – 5,312,690 | **F_420_ dependent reductase** |  |
| **FDR-AA** | MSMEG_1980 | 2,061,887 – 2,063,062 | Hypothetical protein | None |
|  | **MSMEG_1981** | 2,063,059 – 2,063,547 | **FDR-AA enzyme** |  |
|  | **MSMEG_1077** | 1,141,706 – 1,142,155 | **FDR-AA enzyme** |  |
|  | MSMEG_1078 | 1,142,177 – 1,142,866 | Hydrolase |  |
|  | MSMEG_3202 | 3,281,139 – 3,282,659 | Hypothetical protein |  |
|  | MSMEG_3203 | 3,283,047 – 3,283,682 | Transporter LysE family protein |  |
|  | **MSMEG_3204** | 3,283,704 – 3,284,072 | **FDR-AA enzyme** |  |
|  | MSMEG_5372 | 5,449,234 – 5,451,729 | Sensor protein KdpD |  |
|  | MSMEG_5373 | 5,451,846 – 5,452,871 | Nitrilase 2 |  |
|  | MSMEG_5374 | 5,452,917 – 5,454,350 | Glutamate- ammonia ligase |  |
|  | MSMEG_5375 | 5,454,493 – 5,455,191 | GntR family transcriptional regulator |  |
|  | **MSMEG_5376** | 5,455,289 – 5,455,684 | **FDR-AA enzyme** |  |
|  | MSMEG_5377 | 5,456,127 – 5,456,711 | Hypothetical protein |  |
|  | **MSMEG_0966** | 1,039,684 – 1,040,067 | **FDR-AA enzyme** |  |
|  | **MSMEG_0967** | 1,040,090 – 1,040,470 | **FDR-AA enzyme** |  |
| **FDR-B subclade** | MSMEG_6843 | 6,892,736 – 6,893,557 | NAD-dependent epimerase/dehydratase | None |
|  | MSMEG_6844 | 6,893,571 – 6,894,623 | trap-t family protein  transporter dctp |  |
|  | MSMEG_6845 | 6,894,636 – 6,895,931 | trap dicarboxylate  transporter dctm subunit |  |
|  | MSMEG_6846 | 6,895,944 – 6,896,453 | C4 decarboxylate  transporter |  |
|  | MSMEG_6847 | 6,896,461 – 6,897,615 | Hypothetical protein |  |
|  | **MSMEG_6848** | 6,897,798 – 6,899,045 | Oxidoreductase, **F_420_**  **dependent reductase** |  |
|  | MSMEG_6849 | 6,899,091 – 6,900,023 | LysR family transcriptional  regulator |  |

^1.^ Double lines are used to separate different operons and the F_420_ dependent reductases are in bold.
